# Vitamin D Improves Neurogenesis and Cognition in a Mouse Model of Alzheimer’s Disease

**DOI:** 10.1007/s12035-017-0839-1

**Published:** 2018-01-09

**Authors:** Maria Morello, Véréna Landel, Emmanuelle Lacassagne, Kevin Baranger, Cedric Annweiler, François Féron, Pascal Millet

**Affiliations:** 1 Aix Marseille Univ, CNRS, NICN, Marseille, France; 2 Clinical Biochemistry, Department of Experimental Medicine and Surgery, Faculty of Medicine, University Hospital of Tor Vergata, Rome, Italy; 3 Division of Clinical Nutrition and Nutrigenomics, Department of Biomedicine and Prevention Faculty of Medicine, University of Tor Vergata, Rome, Italy; 4 Department of Neurosciences and Aging, Division of Geriatric Medicine, Angers University Hospital, Angers University Memory Clinic, Research Center on Autonomy and Longevity, UPRES EA 4638, University of Angers, UNAM, Angers, France; 5 Robarts Research Institute, Department of Medical Biophysics, Schulich School of Medicine and Dentistry, The University of Western Ontario, London, ON Canada

**Keywords:** Vitamin D supplementation, Vitamin D deficiency, Alzheimer’s disease, Mouse model, In vitro and in vivo neurogenesis, Memory, Sexual dimorphism

## Abstract

The impairment of hippocampal neurogenesis at the early stages of Alzheimer’s disease (AD) is believed to support early cognitive decline. Converging studies sustain the idea that vitamin D might be linked to the pathophysiology of AD and to hippocampal neurogenesis. Nothing being known about the effects of vitamin D on hippocampal neurogenesis in AD, we assessed them in a mouse model of AD. In a previous study, we observed that dietary vitamin D supplementation in female AD-like mice reduced cognitive decline only when delivered during the symptomatic phase. With these data in hand, we wondered whether the consequences of vitamin D administration on hippocampal neurogenesis are stage-dependent. Male wild-type and transgenic AD-like mice (5XFAD model) were fed with a diet containing either no vitamin D (0VD) or a normal dose of vitamin D (NVD) or a high dose of vitamin D (HVD), from month 1 to month 6 (preventive arm) or from month 4 to month 9 (curative arm). Working memory was assessed using the Y-maze, while amyloid burden, astrocytosis, and neurogenesis were quantified using immunohistochemistry. In parallel, the effects of vitamin D on proliferation and differentiation were assayed on primary cultures of murine neural progenitor cells. Improved working memory and neurogenesis were observed when high vitamin D supplementation was administered during the early phases of the disease, while a normal dose of vitamin D increased neurogenesis during the late phases. Conversely, an early hypovitaminosis D increased the number of amyloid plaques in AD mice while a late hypovitaminosis D impaired neurogenesis in AD and WT mice. The observed in vivo vitamin D-associated increased neurogenesis was partially substantiated by an augmented in vitro proliferation but not an increased differentiation of neural progenitors into neurons. Finally, a sexual dimorphism was observed. Vitamin D supplementation improved the working memory of males and females, when delivered during the pre-symptomatic and symptomatic phases, respectively. Our study establishes that (i) neurogenesis is improved by vitamin D in a male mouse model of AD, in a time-dependent manner, and (ii) cognition is enhanced in a gender-associated way. Additional pre-clinical studies are required to further understand the gender- and time-specific mechanisms of action of vitamin D in AD. This may lead to an adaptation of vitamin D supplementation in relation to patient’s gender and age as well as to the stage of the disease.

**Electronic supplementary material:**

The online version of this article (10.1007/s12035-017-0839-1) contains supplementary material, which is available to authorized users.

## Background

Vitamin D, a well-known seco-steroid hormone, has been increasingly implicated in the pathophysiology and the progression of many neurological diseases, including Alzheimer’s disease (AD) [1–4]. As the world faces a pandemic AD, this overlooked link should be further examined to allow a deeper understanding of the complex pathophysiology of the disease and potentially enrich the disappointing current therapeutic arsenal.

Calcitriol (1,25(OH)2D3), the active form of vitamin D, operates via a vitamin D receptor (VDR) (part of the superfamily of steroid hormone receptors) that interacts with specific genomic sequences (above 1000) named vitamin D responsive elements (VDREs) found in promoter regions [5–7]. Epidemiologic and genetic studies indicate that AD is associated with hypovitaminosis D [8–10], VDR polymorphisms [11–14], and dysregulated VDR mRNA [15]. Very recently, an interventional study revealed that vitamin D modulates the serum level of the beta amyloid peptide Aβ1–40 in AD patients, suggesting an improved Aβ clearance [16].

In various mouse models of AD, a vitamin D supplementation diminishes the amyloid burden [17–19] and increases Aβ clearance by the blood-brain barrier [20, 21]. Consistently, a vitamin D deficiency worsens cognition in a rat model of AD [22] and increases Aβ production via a modulation of APP processing in wild-type (WT) animals [23]. Moreover, it has been observed that (i) an extended number of AD-related genes present a VDRE in their coding or non-coding sequences [24], (ii) a prenatal vitamin D depletion induces a dysregulated expression of AD-related genes [25, 26], and (iii) the transgenic 5XFAD mouse model of AD [27] displays a timely dysregulated expression of hundreds of vitamin D-related genes [28]. Lastly, a vitamin D-regulated production of Aβ 1–42 occurs in primary cultures of cortical neurons [29].

Such converging evidence led us to assess the effects of an early or late cholecalciferol (pre-vitamin D) supplementation on the course of the pathology. Female wild-type and 5XFAD mice were fed or not with a vitamin D-enriched diet, from month 4 to month 9. An improved learning and memory performance as well as a decrease in amyloid plaques and astrogliosis were noticed in transgenic animals. At the molecular level, the transcriptome of the hippocampus and neocortex of mice at M9 revealed a large panel of dysregulated pathways, and it appeared that vitamin D action engages in the crosstalk with estrogen and insulin signaling [19]. In parallel, we performed a study based on a pre-symptomatic vitamin D supplementation in female 5XFAD mice. Protocols and assessments were identical, the only difference being the timing (from M1 to M5) of the cholecalciferol enrichment. To our surprise, no statistically significant change on cognition and pathological features was observed (unpublished data).

In mammals, neurogenesis participates to cognitive functions throughout life and the neurons newly generated in the hippocampus participate to various forms of learning, memory, mood control, and perception [30, 31]. Yet, adult neurogenesis diminishes throughout lifetime, declining along with cognition [32–34]. Partly linked to the toxicity of β-amyloid peptide on neural stem and progenitor cells, a reduced neurogenesis might also participate to the cognitive impairment in AD [35, 36]. However, data are conflicting since a reduced or an enhanced endogenous neurogenesis was reported [37–39]. Possibly, neurogenesis impairment in AD might be stage-dependent and mainly observed in the early phases of the disease [36].

Vitamin D is suspected to be a potential modulator of neurogenesis. First, a prenatal vitamin D deficiency disrupts brain development and alters the expression of growth factors and neurotrophin receptors in the adult dentate gyrus [40]. Second, vitamin D stimulates the synthesis of NGF within the hippocampus, leading to an enhanced neurite outgrowth and a reduced cellular proliferation [41, 42]. Third, a maternal vitamin D deficiency alters neurogenesis in developing rat brains [43]. Fourth, an adult hypovitaminosis D increases the proliferation of neuroblasts in the sub-granular zone of the hippocampus and alters their neuronal differentiation [44]. However, calcitriol enhances proliferation in secondary cultures of neural progenitor cells [45] and a recent study failed to report any impact on hippocampal proliferation in adult mice [46].

Despite these studies, nothing is known about the impact of vitamin D on hippocampal neurogenesis during aging or in neurodegenerative disorders. To further elucidate the potential roles of cholecalciferol and calcitriol on adult neurogenesis in normal aging and AD, we studied the effect of three diets containing either no vitamin D (0VD) or a normal dose of vitamin D (NVD) or a high dose of vitamin D, delivered from month 1 to month 6 (preventive arm) or from month 4 to month 9 (curative arm), on the proliferation and differentiation of hippocampal neural progenitor cells in hippocampi of 5xFAD mice and their WT counterparts. In parallel, we evaluated the working memory, the amyloid burden, and astrogliosis in animals from both genotypes. We finally assessed the direct effects of calcitriol on proliferation and differentiation in primary cultures of murine neural progenitor cells and neurons.

## Materials and Methods

### Animals and Experimental Design

Male 5XFAD transgenic mice were used for this study. These mice overexpress two transgenes bearing five mutations linked to familial AD: human APP (Swedish mutation K670N, M671L; Florida mutation I716V; London mutation V717I) and human presenilin 1 (PSEN1 M146L, L286V), under transcriptional control of the mouse Thy1 promoter. 5XFAD lines from the B6/SJL genetic background were maintained by crossing hemizygous transgenic mice with B6/SJL F1 breeders. These mice exhibit AD-related symptoms earlier than other animal models, and amyloid deposition starts in the cortex and subiculum at 2 months of age [27]. Heterozygous 5XFAD transgenic animals and wild-type (WT) controls were obtained after breeding of progenitors purchased from the Jackson Laboratory. Newborn pups were genotyped by polymerase chain reaction (PCR) of tail DNA biopsies in order to detect the human PSEN1 gene. Animals were weaned at 4 weeks of age and divided into two arms: the first called “preventive” have been immediately fed from M1 to M6 with one of the three different vitamin D diets described below, and the second one, called “curative” were fed from M4 to M9 with the same diets. Inside preventive or curative arms, mice were allocated to three different groups (*n* = 10 to 14): WT animals on a control diet (NVD = 1000 IU/kg) (INRA, France), WT animals on a vitamin D3-enriched diet (HVD = 7500 IU/kg) (INRA, France), WT animals on a totally vitamin D3-depleted diet (0VD = 0 IU/Kg) (INRA, France), transgenic animals on a control diet (NVD), transgenic animals fed with the vitamin D3-enriched diet (HVD), and transgenic animals receiving a totally vitamin D3-depleted diet (0VD). For a mouse eating 15 g every day, the HVD diet corresponds to a daily dose of 500 UI/kg/day which has been found optimal for nerve repair [47]. Mice were tested at M5 for the preventive group and M8 for the curative group in the Y-maze before BrdU injection and euthanasia at M6 and M9, respectively. Animal experiments were approved by the Ethics Committee of the Medical Faculty of Marseille and were carried out in accordance with the guidelines published in the European Communities Council Directive of November 24, 1986 (86/609/EEC). All efforts were made to reduce animal suffering and the number of mice needed for the study.

### Behavioral Testing: Y-Maze

Spontaneous alternation in the Y-maze was tested according to the following protocol: each mouse was placed in a random arm of the symmetrical Y-maze and was allowed to explore freely through the maze during an 8-min session. The sequence and total number of arms entered was recorded. Arm entry was considered complete when the hind paws were completely in the arm. Experiments were done blind with respect to the genotype and diet of the mice. Washing with water and ethanol was performed between each passage. Percentage of alternation was determined as follows: number of triads containing entries into all three arms/maximum possible alternations (total number of arms entered − 2) × 100.

### BrdU Injection and Tissue Processing

Forty-eight hours before euthanasia, mice were intraperitoneally injected with a BrdU (Sigma) sterile solution (10 mg/ml final in NaCl 0.9%) for a total of 200 mg/kg. Two days later, mice were anesthetized with pentobarbital at 60 mg/kg, intracardially perfused by PBS for 10 min followed by 30 ml of 4%PFA, and brains isolated from each animal. The brains were then postfixed in 4% PFA at 4 °C for 24 h, before being transferred and stored in 4 °C cold PBS. Brains were finally transferred in 4 °C cold 30% sucrose solution for 24 h before being snap frozen in − 80 °C cooled isopenthane and stored dry at − 80 °C. Coronal 40-m-thick brain sections were obtained by using a cryostat (Leica-Microsystems) and stored at − 20 °C in six-well plates containing a cryoprotectant solution (30% ethylene glycol, 30% glycerol (Sigma-Aldrich, Saint-Quentin Fallavier, France), in 0.05 M phosphate-buffered saline PBS (pH 7.4)) until being processed for immunostaining. Coronal floating sections through the hippocampus containing the entire dentate gyrus between bregma − 1.34 mm and bregma − 3.28 mm (Paxinos and Watson 1998 atlas of mouse brain) were used for immunohistochemistry procedures.

### Immunostaining of Tissues

For Aβ, GFAP, and DCX staining on brains, after washing in PBS, four free-floating sections (every six serial sections) of each brain from similar regions were incubated in a PBS blocking solution containing 3% BSA and 0.1% Triton X-100 followed by overnight incubation at 4 °C with mouse monoclonal anti-Abeta 6E10 1:300 (Covance Eurogentec, France), rabbit polyclonal anti-GFAP 1:500 (Dako France, Trappes, France), and rabbit polyclonal anti-DCX 1:250 (Abcam, France). Then, slices were rinsed in PBS and incubated for 90 min at RT with cross-absorbed Alexafluor 488-conjugated anti-rabbit or 594-conjugated anti-mouse secondary antibodies (1/500, Life Technologies, Saint Aubin, France) along with Hoechst blue (1/1000, Sigma-Aldrich) in dark conditions. After several washes in PBS, slides were mounted with ProLong Gold Antifade reagent (Life Technologies). Omission of primary antibodies was used as negative controls of immunostaining. For BrdU immunolabeling, the slides were incubated for 2 h at 65 °C in 50% formamide (Sigma) in PBS. After, the slides were washed for a total of 15 min with PBS. The sections were treated with 2 N HCL (30 min at 37 °C) and then rinsed in borate buffer for 10 min (0.1 M pH 8.4). The sections were washed with PBS for 1 h and then incubated with PBS containing 0.3% Triton X-100 and 5% normal goat serum for 2 h at room temperature (blocking solution). The sections were then incubated in monoclonal rat anti-BrdU FITC combined (1/200 Abcam) diluted in PBS containing 0.3% Triton X-100 and 1% normal goat serum at 4 °C for 24 h under agitation. All the sections incubated were rinsed several times in PBS and then incubated under agitation for 2 h. Omission of primary antibodies was used as negative controls of immunostaining. Finally, all sections were incubated for 90 min at RT with 0.5 μg/ml DNA intercalating Hoechst (Life Technologies) in dark conditions. After several washes in PBS, the slides were mounted with ProLong Gold Antifade reagent (Life Technologies).

### Microscopic Analysis and Quantification of Brain Sections

Four sections (every six serial sections) of each brain from similar regions containing the right and left of cortex and hippocampus (between bregma − 1.34 mm and − 3.28 mm-ref. Paxinos and Watson atlas) were observed by two investigators under Axiovent inverted microscope equipped with DAPI, FITC, and rhodamine epifluorescence filters (Zeiss, Jena, Germany). The deposition of amyloid plaques and glial reaction in cortex and hippocampus were performed in the same sections. The total number of Aβ plaques was manually counted, with investigators blinded to conditions, and the intensity of GFAP positive glial reaction was measured by Axiovision software (Zeiss). Quantification of neurogenesis, by counting of BrdU+ and DCX+ cells, was performed in hippocampal formation at the level of the dentate gyrus (DG). The total numbers of BdU+ and DCX+ cells were manually counted, blind of the conditions for the investigators, at × 20 magnification in four hippocampi for each animal. All the measurements were performed on images of large brain sections (contained cortex and hippocampus) obtained using the mosaic mode of Axiovision. The area of each cortex and hippocampus were measured at × 20 magnification. Results of density of neurons and Abeta plaques were expressed as the average number/area mm^2^ and the intensity of GFAP reaction were expressed as mean total intensity/mm^2^ (cortex and hippocampus). The entity of neurogenesis was evaluated quantified the total numbers of BdU+ and DCX+ per hippocampi.

### In Vitro Experiments

#### Primary Mouse Embryonic Cortical Cell Cultures and Vitamin D Treatment

Wild-type mice were used for cortical preparations and primary cultures were established from E14.5 fetus. Days of gestation were calculated from plug observation. Cortices were dissected in 0.6% glucose-containing PBS (Sigma) under a binocular microscope. Cleared of the meninges, the cortices and then the cells were gently mechanically dissociated by a succession of pipetting actions, before being centrifuged at 300 rpm for 10 min. For the adherent neural progenitors, the cells were plated on poly-d-lysine-coated glass coverslips in 12-well plates at 0.5 × 10^6^ cells/ml in DMEM supplemented with 2% B27 (Invitrogen), penicillin (100 U/ml; Sigma), streptomycin (100 Ag/ml; Sigma), human recombinant FGF2 (20 ng/ml; Sigma), and EGF (20 ng/ml; Sigma). When cultured in suspension (neurospheres), the progenitor cells were grown in the same medium at the same density but in uncoated 12-well plates. All the primary cultures of progenitor cells were cultivated 2 days at 37 °C in a 5% CO_2_ atmosphere before calcitriol treatment. For the neuronal primary cultures, the E14.5 progenitors were plated at 0.25 × 10^6^ cells/ml in a differentiation-inducing medium: DMEM supplemented with 2% fetal calf serum (Sigma), 2% B27 (Invitrogen), penicillin (100 U/ml; Sigma), and streptomycin (100 Ag/ml; Sigma). Two hours later, the medium was replaced by a growth factor-free medium-DMEM supplemented with 2% B27 (Invitrogen), penicillin (100 U/ml; Sigma), and streptomycin (100 Ag/ml; Sigma) and cultivated 3 days, at 37 °C in a 5% CO_2_ atmosphere.

For vitamin D3 treatment, a stock solution of calcitriol (Sigma-Aldrich) was prepared at 200 mM in absolute and filtered alcohol (Sigma-Aldrich). Ten liters of aliquots were stored at − 20 °C and used rapidly on ice and out of light, maximum twice to avoid concentration variations due to ethanol evaporation.

The adherent and the neurosphere progenitors were treated by calcitriol, 48 h after plating. The progenitor culture treatment medium was prepared as described above and divided into two large 50 or 14-ml Falcon tube in which cold calcitriol was added at the final concentration of 100 M. Tubes were shaken and incubated 10 min at 37 °C to ease ethanol evaporation in both calcitriol-containing and vehicle media. Primary cultures of progenitors were then treated by either calcitriol-containing or vehicle medium and cultivated for three additional days at 37 °C in a 5% CO_2_ atmosphere, before fixation in 4% PFA and storage at 4 °C in PBS.

The cultivated neurons were treated 72 h after plating. The treatment media were prepared as described above in the neuronal growth factor-free medium. Primary neuronal cultures were then treated by either calcitriol-containing (100 M) or vehicle medium and cultivated seven additional days, at 37 °C in a 5% CO_2_ atmosphere, with a replacement of treatment medium every 3 days.

#### Immunostaining of Primary Cultures

Fixed cells were rinsed with PBS, permeabilized with a solution of 0.1% Triton-X100 and 0.1% sodium citrate, and incubated in a PBS blocking solution, containing 3% BSA (Sigma) and 0.1% Triton X-100 (Sigma), followed by a 2-h incubation at room temperature under agitation with a rabbit polyclonal anti-Ki67 1:200 (Abcam, France). After three rinses in PBS, the cells were incubated with a fluorescent green goat anti-rabbit Alexa Fluor 488 (Life Technologies, France) diluted at 1:400. The cells were then incubated 5 min with 0.5 g/ml DNA intercalating Hoechst (Life Technologies), before being rinsed and mounted using Prolong Gold Antifade reagent (Life Technologies). For MAP2 and nestin staining, the cells were immunolabeled as described above. The primary antibody solutions used were as follows: a polyclonal chicken anti-MAP2 (1/200, Abcam) and a monoclonal mouse anti-nestin (1/200, Abcam). After three rinses in PBS, the cells were incubated in a fluorescent goat red anti-rabbit Alexa Fluor 594 (Life Technologies, France) and green goat anti-mouse Alexa fluor 488 (Life Technologies, France) diluted at 1:400. The cells were then incubated 5 min at RT with 0.5 g/ml DNA intercalating Hoechst (Life Technologies) before being rinsed and mounted using Prolong Gold Antifade reagent (Life Technologies).

#### Microscopic Analysis and Quantification of Primary Cultures

For primary cell cultures, 7 to 12 independent randomly pictures of every condition were taken under an Axiovent inverted microscope equipped with DAPI, FITC, and rhodamine epifluorescence filters (Zeiss, Jena, Germany). Counting was semiautomated on seven fields/condition minimum under ImageJ, using both automatic thresholding and cell counting for KI67, MAP2, and nestin and manual counting for Hoechst blue stained nuclei. Three independent experiments (> 2000 cells analyzed per condition and experiment for neurons and > 10,000 cells for neural progenitors) were quantitated.

#### Western Blots

Quantitative Western blots were performed for PCNA expression in primary cultures of neural progenitors. Briefly, 72 h after calcitriol treatment, the cells were harvested in PBS and pelleted after centrifugation (300 g for 10 min at RT). Cell pellets were resuspended in 25% *w*/*v* of 50 mM Tris-HCl buffer, pH 7.5 containing 2% SDS, then sonicated and centrifuged at 10,000*g* for 10 min at 4 °C. Protein concentrations were determined using a Bio-Rad DCTM protein assay kit (Bio-Rad, France) and 50 g of protein was run on 10–15% SDS-PAGE gels and transferred onto nitrocellulose membranes (Amersham Bioscience, France). Blots were blocked overnight at 4 °C with 5% non-fat dry milk dissolved in 20 mM Tris-HCl, pH 7.4, 150 mM NaCl and 0.5% Tween 20. PCNA expression was detected by incubation with a monoclonal antibody anti-PCNA (Abcam, 1:1000) and then incubated with a secondary IgG antibody. PCNA-immunoblot signals were visualized using the ECL chemiluminescence kit (GE Healthcare, France) and scanned and quantified using the ImageJ software. Results are representative of three independent experiments.

#### Cell Cycle Analysis

Adherent primary neural progenitor cells were collected by trypsination (60 s of 37 °C warmed 1/10 trypsin solution in PBS), blocked with a 37 °C warmed 1/10 FCS solution, gently pipetted and centrifuged at 300 rpm for 10 min. The pellets were snap fixed in − 20 °C cold 80% ethanol (Sigma) and stored at − 20 °C ≥ 24 h. The day of analysis and after PBS washings, cells were resuspended in PBS containing 50 Ag/ml PI and 10 Ag/ml RNase–DNase-free (Sigma). The cell suspension was kept 30 min at room temperature, and then, cell cycle distribution was measured by flow cytometry on FACSCanto-II flow cytometer (BD Biosciences, France) equipped by BDFACS-Diva software A+.

Neurospheres of primary cultures of neural progenitors were dissociated without trypsination, by gentle pipetting, fixed as described above, and stored at − 20 °C ≥ 24 h. Fixed cell suspensions were then stained and their distribution in the cell cycle measured as described above. At least three independent experiments were performed for the cell cycle analysis.

#### RNA Isolation

Total RNA was isolated from the snap-frozen pellets of 3- day-old primary cultures of non-adherent neural progenitor cells in neurospheres, obtained from E14.5 WT mice embryos, and 10-day-old primary cultures of neurons, issued from the differentiation of E14.5 WT mice embryos. We used RNeasy Mini kit (Qiagen, Courtaboeuf, France), according to the manufacturer’s instructions. RNA concentration was determined using a Nanodrop 2000 spectrophotometer (Life Technologies ThermoFisher Scientific, Villebon sur Yvette, France) and RNA integrity assessed on an Agilent 2100 Bioanalyzer (Agilent Technologies, Les Ulis, France).

#### Real-Time Quantitative PCR (qPCR)

Total RNA (750 ng) was subjected to reverse transcription reaction to synthetize cDNA using oligo dT, RNase Out, and M-MLV RT enzyme (Life Technologies, ThermoFisher Scientific), according to the manufacturer’s instructions. Real-time qPCR experiments were carried out with Sybr Green ITaq (Universal Sybr Green super mix). Gene expression assay: VDR (VDR For 5-TGA CCC CAC CTA CGC TGA CT-3′, VDR Rev 5′-CCT TGG AGA ATA GCT CCC TGT ACT-3′). Experiments used 25 ng of previously prepared cDNA, and samples were run in triplicate on six different biological samples for each group. Relative expression levels were determined according to the ΔΔCt method where the expression level of the mRNA of interest is given by 2^−ΔΔCT^ where ΔΔCT = ΔCT target mRNA−ΔCT reference mRNA. Gene expression assay: HPRT (HPRT For 5′-GCT CGA GAT GTC ATG AAG GAG A-3, HPRT Rev 5′-TCA GCG CTT TAA TGT AAT CCA GC-3′) in the same sample as previously described [19].

#### Statistical Analysis

We used one-way ANOVA followed by a post hoc Fisher’s LSD test for multiple comparisons. Kruskal-Wallis test was used to compare two experimental groups. Values represent the mean ± SEM of the indicated number of independent experiments/animals, and the level of significance was set for *p* < 0.05* or *p* < 0.01**.

##### Data Availability

Materials described in the manuscript, including all relevant raw data, are freely available to any scientist wishing to use them for non-commercial purposes.

## Results

### Vitamin D Improves Working Memory of Transgenic Mice Only When Delivered During the Early Stages of the Disease

**Fig. 1:**
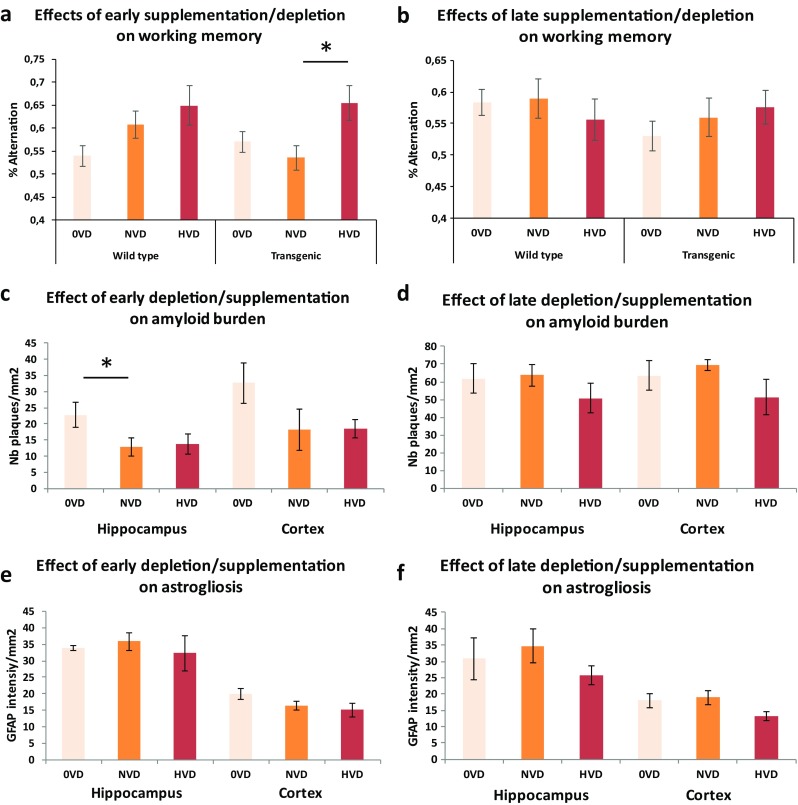
In vivo effect of early (M1 to M5) or late (M5 to M9) vitamin D depletion/supplementation on working memory, amyloid burden, and astrogliosis. A 4-month cholecalciferol supplementation improves memory when delivered during the presymptomatic (**a**) but not during the symptomatic (**b**) phase (*n* = 8–12). A 4-month cholecalciferol depletion increases the amyloid load in wild-type and transgenic animals when set up during the presymptomatic phase (**c**) and not the symptomatic phase (**d**) (*n* = 6). Cholecalciferol depletion/supplementation does not alter astrogliosis in both wild-type and transgenic mice (**e**, **f**) (*n* = 6). *p < 0.05. Diets: 0VD = no vitamin D; NVD = normal dose of vitamin D; HVD = high dose of vitamin D

Working memory was assessed using a Y-maze apparatus. In wild-type animals, vitamin D depletion during young age is not associated with a reduced working memory when compared with a high supplementation. Such an effect is absent in older wild-type mice. Mice fed with a vitamin D-depleted (0VD), vitamin D control (NVD), or vitamin D-supplemented (HVD) diet, from M1 to M5 (Fig. 1a) or from M4 to M8 (Fig. 1b), display the following percentage of alternation: 0.539 ± 0.022, 0.607 ± 0.029, and 0.648 ± 0.039 (preventive arm), 0.583 ± 0.020, 0.588 ± 0.031, and 0.556 ± 0.032 (mean ± SEM) (curative arm), respectively. In transgenic animals, a high vitamin D supplementation significantly improves (*p* < 0.05) working memory only when administered during the first stages of the disease (Fig. 1a). For the 0VD, NVD, and HVD groups, from M1 to M5 (Fig. 1a) or from M4 to M8 (Fig. 1b), the percentage of alternation are 0.570 ± 0.022, 0.535 ± 0.026, 0.655 ± 0.038, 0.530 ± 0.023, 0.560 ± 0.030, and 0.575 ± 0.026 (mean ± SEM), respectively.

### The Time-Dependent Action of Vitamin D Varies According to the Gender

In order to assess a putative gender dimorphism, we assessed the working memory of male and female transgenic mice fed with two diets (NVD and HVD) during the same time windows. Cholecalciferol supplementation distinctively affects cognitive outcome in male and female mice [see Additional file 1]. For the female mice fed with a NVD or HVD diet, from M1 to M5 (A) or from M4 to M8 (B), the percentage of alternation are 64 ± 04, 66 ± 03, 47 ± 02, 60 ± 03 (mean ± SEM), respectively. For the male mice fed with a NVD or HVD diet, from M1 to M5 (C) or from M4 to M8 (D), the percentage of alternation are 54 ± 03, 65 ± 04, 56 ± 03, 58 ± 03, respectively.

### Vitamin D Depletion during the Early Stages Worsens the Hippocampal Amyloid Load

Vitamin D supplementation betters working memory in AD mice at early stages. However, such an improvement is not associated to a reduced amyloid burden or astrogliosis, as observed on Fig. 1c–f. Conversely, vitamin D deficiency at an early stage induces a significantly increased number of amyloid plaques in the hippocampus and the cortex (Fig. 1c), such an effect being absent at a later stage. Transgenic mice fed with a vitamin D-depleted (0VD), vitamin D control (NVD), or vitamin D-supplemented (HVD) diet, from M1 to M6 (Fig. 1c) or from M4 to M9 (Fig. 1d), display, in the hippocampus and the cortex, the following numbers of plaques/mm^2^: 23 ± 04, 13 ± 03, 14 ± 03, 33 ± 06, 18 ± 06, and 18 ± 02 (preventive arm) and 62 ± 08, 63 ± 06, 51 ± 08, 64 ± 08, 69 ± 03, and 51 ± 09 (curative arm).

Astrogliosis is usually associated with the progression of Aβ production and inflammation in the 5xFAD animal model. However, Fig. 1e, f indicates that astrocyte inflammation remains steady between M6 and M9. In addition, vitamin D depletion or supplementation fails to alter this pathological feature.

### An Early High Vitamin D Supplementation Improves Neurogenesis in the Hippocampus of Transgenic Animals

**Fig. 2:**
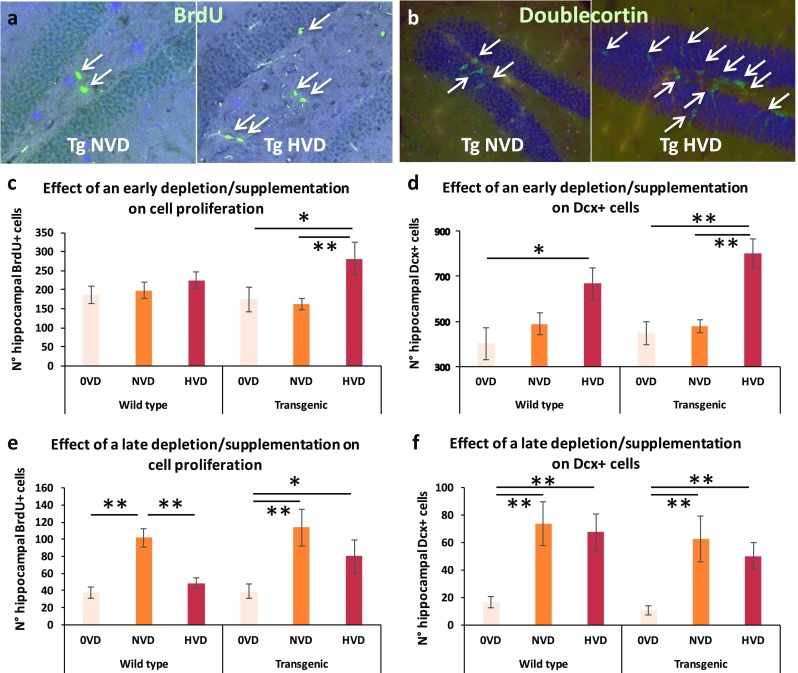
In vivo effect of early (M1 to M5) or late (M5 to M9) vitamin D depletion/supplementation on neurogenesis in the dentate gyrus. Neurogenesis was assessed by quantifying the number of BrdU-positive (**a**) and doublecortin-positive cells (**b**) in male mice. A 4-month cholecalciferol supplementation, during the presymptomatic phase, improves cell proliferation (**c**) and neural progenitor cell proliferation and/or differentiation (**d**) in transgenic mice (*n* = 8–12). A 4-month cholecalciferol depletion, during the symptomatic phase, reduces neurogenesis in transgenic mice (**e**, **f**) (*n* = 6). *p < 0.05; **p < 0.01. Diets: 0VD = no vitamin D; NVD = normal dose of vitamin D; HVD = high dose of vitamin D

Hippocampal neurogenesis decreases with normal aging and in early phases of AD. We assessed hippocampal neurogenesis in our animals by the evaluation, in the sub-granular zone (SGZ) of the dentate gyrus, of both proliferation and differentiation of neural stem and progenitor cells by quantifying BrdU incorporation and doublecortin (DCX) expression [48–51]. An early high vitamin D supplementation significantly triggers cell proliferation in the dentate gyrus of transgenic animals (Fig. 2c), leading to an enhanced neural progenitor cell production and differentiation (Fig. 2d). In wild-type mice, the number of DCX-expressing cells is also increased (Fig. 2d) although cell division is not amplified (Fig. 2c). Wild-type and transgenic mice fed with a vitamin D-depleted (0VD), vitamin D control (NVD), or vitamin D-supplemented (HVD) diet, from M1 to M6, display the following numbers of BrdU-positive cells/mm^2^—54.600 ± 5.185, 55.166 ± 6.838, 64.800 ± 8.398, 47.000 ± 7.545, 49.166 ± 4.028, and 81.500 ± 11.017 (Fig. 2c)—and doublecortin-positive cells—117.333 ± 19.590, 135.600 ± 10.386, 187.166 ± 25.564, 124.500 ± 10.350, 145.833 ± 9.228, and 230.200 ± 19.685 (Fig. 2d), respectively.

### A Late Vitamin D Depletion Strongly Impairs Neurogenesis in both Wild-Type and Transgenic Animals

Neurogenesis is impaired in aging animals, as observed in Fig. 2e, f. At 9 months of age, the proliferation and the number of DCX-expressing cells in the dentate gyrus of wild-type mice are dramatically reduced (two- and sixfold changes, respectively), when compared with 6-month-old animals (Fig. 2c, d). In our 5xFAD model, at that age, hippocampal neurogenesis is not affected. Cell proliferation and production of neural progenitor cells and immature neurons in the dentate gyrus of transgenic animals remain similar to those observed in wild-type mice.

Interestingly, at that later stage and age, high vitamin D supplementation fails to increase stem or progenitor cell proliferation and neurogenesis, in both WT and 5xFAD mice. Conversely, vitamin D depletion strongly impairs cell proliferation and neuron production and differentiation. As a consequence, the highest level of cell proliferation and the largest DCX population is encountered in the NVD fed groups. Wild-type and transgenic mice fed with a vitamin D-depleted (0VD), vitamin D control (NVD), or vitamin D-supplemented (HVD) diet, from M4 to M9, display the following numbers of BrdU-positive cells/mm^2^—12.2 ± 1.714, 30.6 ± 2.541, 15.6 ± 2.204, 11.8 ± 2.154, 33.2 ± 6.191, and 26.8 ± 6.094 (Fig. 2e)—and doublecortin-positive cells—2.881 ± 1.288, 10.663 ± 4.768, 9.154 ± 4.093, 2.302 ± 1.029, 10.644 ± 4.760, and 6.534 ± 2.922 (Fig. 2f), respectively.

### In Vitro, Calcitriol Increases Proliferation of Primary Cultures of Neural Progenitor Cells

**Fig. 3:**
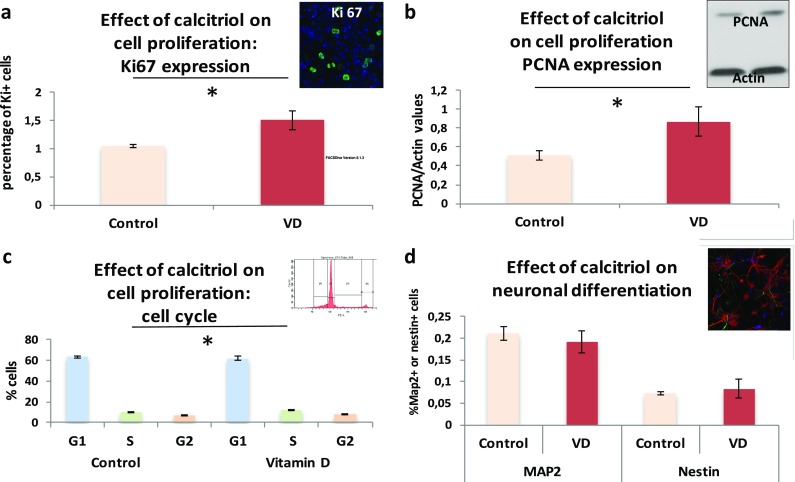
In vitro effect of calcitriol on proliferation and differentiation of neural progenitors and neurons. **a**–**c** E14.5 murine neural progenitor cells were cultivated with or without calcitriol (*n* = 3), during 3 days. Cell proliferation was then quantified using immunochemistry (**a**), Western blotting (**b**), and flow cytometry (**c**). Calcitriol significantly increases the percentage of Ki67-positive cells (**a**), the ratio PCNA/actin (**b**), and the proportion of cells in S phase (**c**). Influence of calcitriol on neuronal differentiation was assessed using primary cultures of neurons. The calcitriol added during 7 days to the culture medium had no effect on the percentage of MAP2+ or nestin+ cells (**d**). *p < 0.05

Vitamin D is known as an antiproliferative agent on immortalized embryonic hippocampal cells [41, 42]. However, it has been recently reported that vitamin D stimulates proliferation in neural stem cell cultures [45]. To assess whether vitamin D exerts a direct effect on neural stem or progenitor cell proliferation, we used another in vitro model of primary cultures of neural progenitor cells. Calcitriol, the active form of vitamin D, was added for 3 days to a culture of neural progenitor cells originating from E14.5 WT mice embryos. Figure 3a–c indicates that vitamin D activates cell proliferation. The normalized number of Ki-67-positive cells in untreated and treated wells is 1.042 ± 0.022 and 1.504 ± 0.163, respectively (Fig. 3a). This finding was confirmed by another experiment based on PCNA expression (Fig. 3b). The ratio PCNA/actin in untreated and treated wells is 0.512 ± 0.049 and 0.871 ± 0.154, respectively. In addition, using flow cytometry, we quantified the number of cells in each phase of the cell cycle. Figure 3c shows that an augmented quantity of cells is observed in S phase, when they are treated with calcitriol. The percentage of cells in the subG1, G1, S, and S2 phases, in untreated and treated wells, is 12.966 ± 0.778, 63.3 ± 1.391, 9.9 ± 0.449, and 6.9 ± 0.205 (untreated) and 12.166 ± 0.897, 62.167 ± 2.148, 12.433 ± 0.801, and 7.9 ± 0.385 (treated), respectively.

### Neuronal Differentiation Is Not Improved by Calcitriol Treatment

Vitamin D supplementation might trigger neuronal differentiation of embryonic stem cells [42] or neural/oligodendrocyte differentiation in secondary cultures of neural stem cells [45]. To assess whether vitamin D can play a direct role on neuronal differentiation or not, we tested the effect of calcitriol supplementation on neuronal differentiation of primary cultures of neuroblasts. Figure 3d indicates that neuronal differentiation is not enhanced by the addition of vitamin D in the differentiating culture medium during 7 days. Likewise, the percentage of nestin-expressing stem cells remains unchanged. The proportion of MAP2-positive and nestin-positive cells in untreated and treated wells is 21.173 ± 1.576 and 19.219 ± 2.592 (MAP2), 7.28 ± 0.364, and 8.341 ± 2.184 (nestin).

To assess whether vitamin D can directly trigger neuronal differentiation or not, we tested the effects of calcitriol on neural progenitor cells cultivated in neurospheres. When undergoing a differentiation process, progenitor cells modify their shape, flatten and adhere to the support of culture, and exhibit a G1 cell cycle arrest [52]. Three days of calcitriol treatment failed in modifying neurosphere shape or their cell cycle, suggesting an absence of neuronal pro-differentiating direct effect on neural progenitor cells. Finally, we assessed the expression of the VDR in our untreated in vitro model of primary cultures of neuroblasts or neurons by PCR. We observed that the VDR gene was not transcribed in neural progenitor cells or during their neuronal differentiation [see Additional file 2].

## Discussion

Although vitamin D deficiency is considered as a risk factor for memory loss in aging and AD, the effects of hypovitaminosis D or vitamin D supplementation on the functioning of aged and/or AD brains have not been thoroughly investigated in animal models. In parallel, despite the possible interactions between vitamin D and neurogenesis, no study has assessed its putative modulation by vitamin D. To the best of our knowledge, the current study, performed in vivo and in vitro, is the first to assess the outcomes of either a sustained vitamin D depletion or supplementation, during two time windows (precocious/preventive versus late/curative), on neurogenesis in both normal and AD-like mouse brains, at the cellular, histological, and behavioral levels.

Our results indicate that high vitamin D supplementation is efficient in improving working memory and endogenous neurogenesis in AD, only when delivered before the onset of the major symptoms. We also observed that vitamin D deficiency impairs neurogenesis in the brains of both wild type and transgenic animals only when occurring at an advanced age or at late stage of AD. At the cellular level, our in vitro experiments indicate that addition of calcitriol, the active form of vitamin D, has a direct positive effect on proliferation of primary cultures of E14.5 neural progenitor cells but not on neuronal differentiation. Finally, the comparison of the present results with those of a previous study suggests a possible gender effect. In female AD-like mice, a chronic vitamin D supplementation was efficient on working memory and amyloid load, only when administered during the symptomatic phase (curative).

### Vitamin D and Working Memory

Cognitive abilities of the 5XFAD mouse model have been extensively characterized. Progression of memory deficits was measured using fear conditioning, Y/T-maze alternation [27, 53], novel object recognition [53, 54], and the Morris water maze [3, 19] assays. With regard to the Y-maze test, a reduced working memory is observed in over 6-month-aged transgenic mice when compared with wild-type animals [55]. However, this impaired working memory is not observed in our cohort. At 5 and 8 months of age, the spontaneous alternation of transgenic mice in a Y-maze was not altered, in comparison with wild-type animals of the same age. Yet, a trend towards a reduction in the 5-month-old 5xFAD group suggests that this lack of effect might be linked to the size of the population studied.

Notwithstanding, we were able to quantify vitamin D-associated changes. A high level of vitamin D induces a significantly improved working memory in 5-month-old transgenic mice when compared with same age siblings fed with a control diet. In wild-type animals, a similar phenomenon is observed but only when the high vitamin D group is weighed against the no vitamin D group. These results are in accordance with the picture emerging from the comparison of 7 previous experiments. Indeed, it has been found that vitamin D supplementation (i) enhances cognitive performance in young transgenic animals [17, 18] and (ii) preserves memory abilities in old transgenic mice or aging rats [3] and that hypovitaminosis D reduces spatial memory capabilities in an AD rat model [22]. Inconsistently, vitamin D seems to improve cognition in WT rather than transgenic animals but such discrepancy can easily be explained by the variety of protocols [3]. Indeed, research teams used various transgenic and non-transgenic male/female/both rodent models of AD, different formulations and doses of vitamin D, diverse ages and time windows for delivery, as well as distinct behavioral assays. Still, all of these studies, including our own, suggest a deleterious effect of hypovitaminosis D and a beneficial effect of vitamin D supplementation on cognition during normal aging and AD in rodent models. This present study and our previous ones [3, 28] advocate for the extension of investigations on humans and animal models of AD before making firm conclusions on the exact conditions where vitamin D might be beneficial. It would also be of great interest, in order to counterbalance the limitations of the Y-maze, to use complementary behavioral assays such as the Morris water or Barnes mazes.

### Vitamin D, Inflammation, and Amyloid Load

AD is an inflammatory pathology, and we have shown that the transgenic 5XFAD mouse model is characterized by the predominance of inflammatory and immune processes [28]. It can therefore be surmised that vitamin D, known to be an immunomodulator [56, 57] reduces inflammation in rodent models of AD. Such an assumption was strengthened by four research teams, including ours, that reported reduced astrogliosis and expression of inflammatory markers in old transgenic animals fed with an above normal dose of vitamin D [17, 19, 58, 59]. However, in the current study, no effect of vitamin D depletion or supplementation on astrogliosis was observed, whatever the time window (preventive or curative) or the strain (wild type or transgenic) considered. This finding is discordant with one of our previous studies showing, in the same animal model with similar time windows and levels of supplementation, that vitamin D reduces astrogliosis [19]. The latter being performed with female mice, we may suspect a gender effect (see below). Two other studies were conducted exclusively with males and reported a diminished expression of GFAP but, on the one hand, another form of vitamin D (D2 instead of D3) was delivered for a longer period (7 months) to a different transgenic mouse model of AD [59] and, on the other hand, vitamin D3 was administered for a very short period of time (3 weeks) to rats that were not engineered to mimic some of the symptoms of AD [17, 58]. Further studies are required to assess the specific in vivo immunomodulatory roles of vitamin D in AD.

Regarding amyloid plaques, the current study failed to reproduce the vitamin D-associated reduction in amyloid load reported by previous studies [17–19, 58, 59]. However, we observed that a presymptomatic hypovitaminosis D leads to an increased number of amyloid plaques in transgenic mice, an outcome partially reported in previous articles studying other animal models of AD [17, 18]. It is now admitted that vitamin D reduces the accumulation of β-amyloid peptide in AD brains by (i) modulating the activity or expression of ADAM10, APP, BACE, Nicastrin, and Presenilin 1/2 [23, 29] and (ii) increasing its clearance by macrophages or through the brain-blood barrier [20, 60]. We can suspect that similar phenomena are at play in the female 5XFAD mouse model, as reported in our previous article [19]. However, it remains to be elucidated why, in male 5XFAD mice, a similar protocol induces a dissimilar result. As recently reported, 1,25(OH)2D3 regulates amyloid processing in a time- and dose-dependent manner [29] and, possibly, males exhibit a different sensitivity to vitamin D. Similarly, it can be expected that vitamin D depletion in males (not tested in 5XFAD female mice) increases the amyloid burden through mechanisms inhibited by a normal dose of vitamin D, at a lower threshold than in females.

### Vitamin D, In Vivo Cell Proliferation, and Neurogenesis

Hippocampal neurogenesis, an element of utmost importance for many cerebral functions, including memory formation [31, 61], is impaired in many animal models of AD [36]. Among the mechanisms involved in a reduced generation of neurons from neural stem cells, we can cite the accumulation of β-amyloid peptides [37, 62–64]. However, in our male 5XFAD mouse model, despite differences in amyloid load, the cell proliferation ratio remained unmodified, when wild-type and transgenic animals were compared. Of note, the issue of endogenous neurogenesis in AD remains debated [36]. The multiple and sometime opposite actions of APP on neurogenesis might explain partly the differences observed between studies [65] and the differences in the time windows studied suggest the possibility that the impact of AD on neurogenesis might be stage-related [36]. For example, it has been reported that the number of newly born doublecortin-positive cells was reduced in the 5XFAD mouse model, between month 2 and month 4, but remained equal between WT and transgenic from month 4 to month 7 [66]. Such a finding on young transgenic AD mice might explain why our older 5xFAD mice, at month 6 and month 9 of age, did not display a significantly different neurogenesis with our WT mice.

Despite these discrepancies and the absence of modified neurogenesis in our male 5XFAD mouse model, we provide here the evidence for the first time that vitamin D modulates hippocampal neurogenesis during both normal aging and in AD condition. The outcome differed according to the time window. When administered during the presymptomatic phase in 5xFAD mice [see Additional file 3], a high vitamin D supplementation improved both stem and progenitor cell proliferation, assessed by BrdU- and DCX-expressing cells representing a differentiated population of neural progenitors, the transiently amplifying progenitors IIb, and early postmitotic immature neurons [51]. Such a result indicates that vitamin D might have, in young 5xFAD males, a beneficial effect on proliferation, differentiation, and early phases of neurogenesis.

On the contrary, in young WT animals, the neurogenesis assessed by DCX-positive population was improved without a significant increase in global proliferation [see Additional file 3]. The observed enrichment of DCX-positive population in early WT might be at least partly considered as an induction of progenitor differentiation, an assumption in phase with the known positive effects of vitamin D on neuronal differentiation in the hippocampus [41].

Later, during the aging process of WT mice or the symptomatic stage of AD, hypovitaminosis D was detrimental to neurogenesis while a normal vitamin D supplementation improved proliferation and the number of newly formed neurons. The reported effects of hypovitaminosis D on hippocampal neurogenesis in WT mice are partly discordant with our own results. A first study showed an enhanced proliferation (yet not translated into enhanced production of functional neurons) in the dentate gyrus of 2-month-old transgenic mice unable to produce calcitriol [44]. A second study observed no modification in proliferation or survival of neurons in the dentate gyrus of 6-month-old wild-type mice [46]. However, due to the lack of data at a more advanced age, the comparison with our results remains limited, leaving open the option that hippocampal cell proliferation is drastically reduced in aged knockout or vitamin D-deprived mice. Our study is therefore the first one to describe hypovitaminosis D, in normal aging and during late stage in 5xFAD mice, as a risk factor for hippocampal neurogenesis.

Interestingly, a normal supplementation in vitamin D, but not a strong one, improved neurogenesis, at an advanced age and late AD stages. This observation, supported by a publication indicating that vitamin D is more efficient in reducing amyloid beta load when at a lower concentration (10^−8^ versus 10^−7^ M) [29], deserves particular attention. Compared with the observation of the precocious group results, it establishes that, at least in our model, the action of vitamin D on neurogenesis appears to be dose- and stage-dependent. This result enlightens the complexity of the potential action of vitamin D on neurogenesis in both normal and AD brains, most probably secondary to the superposition of many different independent or interdependent actions.

Apart from the deleterious effects of amyloid peptides accumulation on neural progenitor cells in AD animal models, other mechanisms are known to impair neurogenesis in AD. An intensified inflammation is one of the most described [67]. Yet, in our 5xFAD male model, vitamin D did not modify significantly these two deleterious phenomena in the neurogenesis modifying vitamin D conditions, if we except the reduced amyloid load in the young NVD treated 5xFAD hippocampi. Therefore, the mechanisms underlying the modulation of neurogenesis in our AD and WT models remain to be elucidated.

**Fig. 4:**
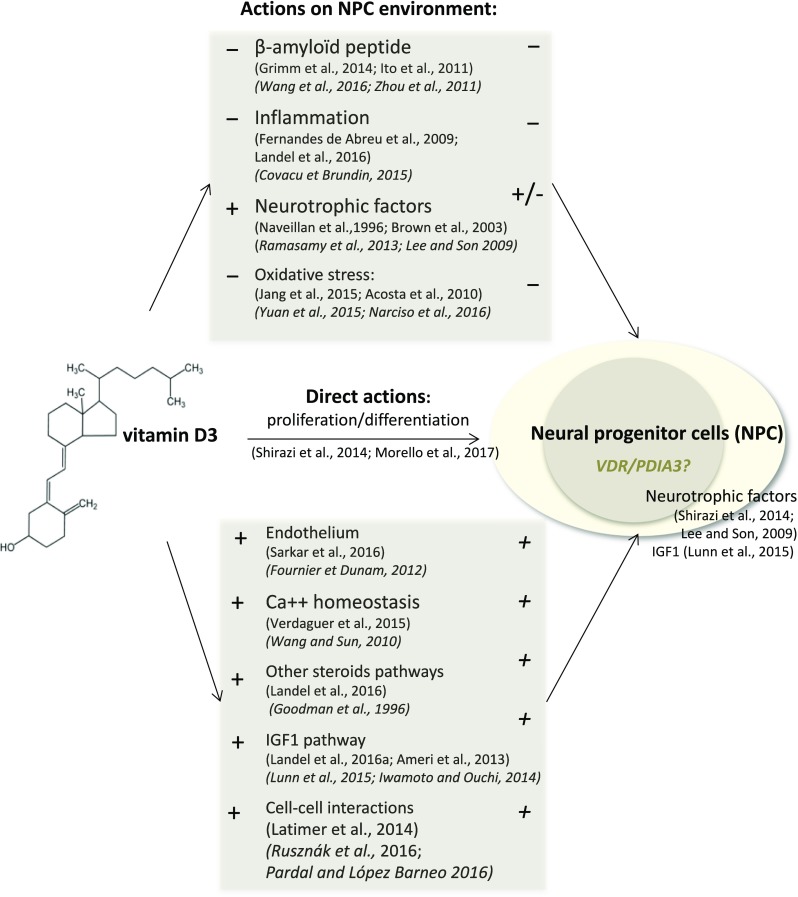
Potential roles of vitamin D on adult hippocampal neurogenesis, during normal aging and in pathological AD condition. The current study established the in vivo link between vitamin D and neurogenesis. The listed studies suggest several direct and indirect actions of vitamin D on neurogenesis. Studies and reviews linking vitamin D3 with biological phenomena or structures are indicated in roman while studies or reviews associating metabolic pathways or structures with neurogenesis are printed in italic. The symbols – and + refer to negative and positive actions, respectively

Several studies allow us to hypothesize that vitamin D might exert, during normal aging or AD, both direct actions on neural stem and progenitor cell biology and indirect actions on their environment (Fig. 4). Most, if not all, of these biological pathways are temporally regulated or modulated during normal aging and in AD. The amyloid deposition and the inflammation reaction are associated to time dependency [27, 68, 69], but also to the production of neurotrophic factors [70, 71], or oxidative stress [72, 73] (Fig. 4).

The differential modulation of these phenomena by vitamin D, during aging or the evolution of the disease, might explain the time-dependent modulation of neurogenesis by vitamin D. The early modulation of neurogenesis by vitamin D is strictly dose-dependent, the higher doses being the most efficient. However, at a later stage, the modulation of neurogenesis by vitamin D is only dose-related, suggesting that the early dose-dependent modulated phenomenon is either non-existent at a later stage, or counteracted by a strictly dose-dependent antagonist mechanism. This beneficial action of vitamin D on neurogenesis, at a late stage and advanced age, should be further investigated in other experiments.

To extend our understanding of the potential in vivo actions of vitamin D on neurogenesis, we decided to study in vitro the possible direct actions of vitamin D on neural progenitor cells. We found that vitamin D might directly stimulate the proliferation in primary cultures of neural progenitor cells, a result consistent with Shirazi et al. However, in our primary cultures of neurons or neurospheres, we failed to observe that vitamin D triggers or enhances neuronal differentiation [see Additional file 3], a finding discordant with those of Shirazi et al. The differences between our two in vitro models might explain this discrepancy. It can also be hypothesized that the potential pro-differentiation effects of vitamin D on neural progenitors in our young WT mice hippocampi is linked to an indirect effect of vitamin D on the environment of the progenitor cells. It is also possible that the in vivo proliferative effects of vitamin D on progenitors can be, at least partly, related to a direct action of vitamin D on the progenitors, in addition to a potential improvement of its environment. However, further studies are necessary before concluding on the possible direct effect of vitamin D on proliferation of neural progenitor cells in vivo. Similarly, we did not observe a spontaneous transcription of VDR in our primary cultures of progenitors or neurons, suggesting that the VDR is not necessarily transcribed during neuronal differentiation in vitro.

The VDR is expressed in several hippocampal areas, especially in the dentate gyrus [74]. Yet, so far, no study has established whether the VDR is expressed in the proliferating cells of the dentate gyrus. Therefore, an in vivo potential VDR-mediated action of vitamin D in stem or progenitor cells remains hypothetical and needs to be studied with appropriate tools. Like other nuclear steroids, vitamin D might exert its effects through both genomic and non-genomic actions [75], especially by membrane receptors such as 1-25D3-MARRS/PDIA3 (membrane-associated rapid response steroid-binding receptor) [54, 76]. Further studies are therefore required to understand whether the direct pro-proliferative and pro-differentiating effects of vitamin D on neural progenitor cells are linked to the presence of the VDR or MARRS/PDIA3 or both. The action of vitamin D on hippocampal neurogenesis during normal aging or in AD might reflect the pleiotropic nature of this steroid hormone.

### A Putative Gender Effect

Recently, our team reported that a high vitamin D supplementation improved cognitive performance in 9-month-old 5XFAD female mice, using both the Y-maze and Morris water maze [19, 28]. This improvement was observed only in the curative preventive group, a result in sharp contrast with the finding reported in the present study. Both studies were based on similar protocols [19], the only exception being the gender of the animals [see Additional file 1]. As a result, the observed differences in working memory appear indicative of a gender effect. Such a sex-specific response to treatments, including vitamin D supplementation, has already been described in other animal models of AD: in 3xTg mice, after an environmental stimulation [77] or an oral vitamin D supplementation [78], in APP/V717I mice treated with geniposide [79] or in APPswe/PS1dE9 mice submitted to prenatal stress [80]. The evolution of AD in male and female seems also to be different in APPswe, PSEN1dE9 mice [81].

The steroid hormone receptor Erα could play important neuroprotective and anti-neuroinflammatory roles in AD [82]. Recently, we have shown that in 9-month-old female 5XFAD mice vitamin D treatment leads to an increased transcription of the gene ESR1 (coding for Erα) and ESR1 related genes [19]. Interestingly ESR1 is implicated in the cleavage of APP [83]. It can be hypothesized that a different gender-related modulation of ESR1 by vitamin D in male and female mice is at play. A modulation of ESR1 expression by testosterone may also be considering since the protective action of testosterone in AD is also mediated by estrogens [84]. Interestingly, during aging, testosterone decreases in male mice and increases in female mice [85, 86]. It can then be surmised that the vitamin D-induced ESR1 transcript expression leads to divergent outcomes in 5XFAD males and females, due to a sex-related production of testosterone.

### The Place of Vitamin D in Clinical Research

Our two recent studies from Landel et al. and the present one suggest that the beneficial effects of vitamin D in an animal model of AD might have some specificities related to the gender of the animal and the stage of the disease. Interestingly, human observational studies underline an association between vitamin D status and cognitive performance, incidence of dementia or AD, which might also be dependent on a variety of factors such as aging or gender. Studies based on woman cohorts report a clear association between hypovitaminosis D and cognitive impairment, which was not always the case in man cohorts [3]. In parallel, results obtained by the Tromso study in 65 years and older patients, revealed that levels of vitamin D appear to be predictive of cognitive outcome only in older individuals [87], suggesting, with other studies, that an age threshold could be at play in the implication of vitamin D in cognitive functions [3]. Longitudinal studies report that hypovitaminosis D even at younger ages is also predictive of AD [10, 88], suggesting that this risk factor might be age independent in humans. Therefore, from a translational point of view, the accumulating amount of evidence coming from experimentation and epidemiology [3] indicate that future clinical studies should be stratified, in order to answer diverse clinically relevant questions such as what kind of vitamin D supplementation, in terms of nature and dose, is more beneficial for AD patients, according to gender, age, disease stage, and type of cognitive impairment. Further studies remain necessary to examine the possible interactions between neuroactive steroid pathways in AD, preferentially preclinical studies on AD brains and prospective clinical studies of potential steroid-linked biomarkers in human fluids.

Some limitations in our study should be acknowledged. The Y-maze is only partially hippocampus-dependent [89]. Furthermore, for a better understanding of the time-dependent neurogenesis, an internal control with a zero time experiment would have been valuable. In addition, an intermediary time point at month 2 would allow to examine the possible vitamin D-associated modulation of the early AD-related impaired neurogenesis in 5XFAD mice. Finally, since normal and pathological aging is generally related to chronic hypovitaminosis D, it would have been interesting to assess the effects of vitamin D supplementation on WT and 5xFAD mice, previously depleted in vitamin D.

## Conclusion

Future studies validating and integrating new tools relative to both neurosteroids and neurogenesis are the next step for deciphering the mechanistic role of vitamin D on neurogenesis. Our study provides additional evidence that vitamin D supplementation may be a simple, efficient, and rationally based therapeutic intervention on the dramatic progression of AD.

## Electronic Supplementary Material

Supplementary Figure 1 In vivo effect of early (M1 to M5) or late (M5 to M8) vitamin D supplementation on working memory, according to the gender. A four-month cholecalciferol supplementation improves memory when delivered during the symptomatic phase in females (A-B) and during the pre-symptomatic phase in males (C-D) (*n* = 8–12). * = *p* < 0.05. Diets: NVD = normal dose of vitamin D; HVD = high dose of vitamin D. (PDF 42 kb)

Supplementary Figure 2 Absence of in vitro effect of calcitriol on the differentiation of neuroblasts (neurospheres). A-B) E14,5 progenitor cells were cultivated in neurospheres, with or without calcitriol (*n* = 4) during 3 days. Cell differentiation was then evaluated by (A) shape modification and neurosphere adhesion to the well and (B) G1 cell cycle arrest during the treatment. C) VDR transcript expression was assessed using quantitative PCR in 3 day-old primary cultures of murine E14,5 progenitor cells and in 10 day-old primary cultures of murine neurons. The VDR is not transcribed neither in neuroblasts nor in neurons. (B, C). Cut off line corresponds to a theoretical DCT for a cDNA amplification at 30 cycles of PCR. (PDF 1436 kb)

Supplementary Table 1 (DOCX 207 kb)

## Vitamin D

1,25(OH)2D3: 1,25-dihydroxycholecalciferol or calcitriol
25(OH)D: 25-hydroxyvitamin D or calcidiol
MARRS: membrane-associated rapid response to steroid
VDR: vitamin D receptor
VDRE: vitamin D responsive element
0VD: no vitamin D
HVD: high dose of vitamin D
NVD: normal dose of vitamin D
PDIA3: protein disulfide isomerase family A member 3

## Animals and Alzheimer’s Disease

5XFAD: 5 mutations of familial Alzheimer’s disease
AD: Alzheimer’s disease
APP: amyloid precursor protein
Aβ: amyloid beta peptide
BACE: beta-secretase
KO: knockout
PSEN1: presenilin 1
TG: transgenic
WT: wild type

## Genes and Proteins

DCX: doublecortin
EGF: epidermal growth factor
ESR1: estrogen receptor 1
FGF2: fibroblast growth factor 2
HPRT: hypoxanthine phosphoribosyltransferase
MAP2: microtubule associated protein 2
NGF: nerve growth factor
PCNA: proliferating cell nuclear antigen

## Protocols

BrdU: bromodeoxyuridine
E14.5: embryonic day 14.5
M1; M4; M5; M6; M8; M9: month 1; month 4; month 5; month 6; month 8; month 9
PBS: phosphate-buffered saline
PCR: polymerase chain reaction
PFA: paraformaldehyde
