# Supplementary material for: Vitamin D Improves Neurogenesis and Cognition in a Mouse Model of Alzheimer’s Disease
Source: Mol Neurobiol. 2018 Jan 9;55(8):6463–79. doi: 10.1007/s12035-017-0839-1 (PMC6061182; doi:10.1007/s12035-017-0839-1)
Supplement: Supplementary file 1 — In vivo effect of early (M1 to M5) or late (M5 to M8) vitamin D supplementation on working memory, according to the gender. A four-month cholecalciferol supplementation improves memory when delivered during the symptomatic phase in females (A-B) and during the pre-symptomatic phase in males (C-D) (n = 8–12). * = p < 0.05. Diets: NVD = normal dose of vitamin D; HVD = high dose of vitamin D. (PDF 42 kb) [file 12035_2017_839_MOESM1_ESM.pdf]

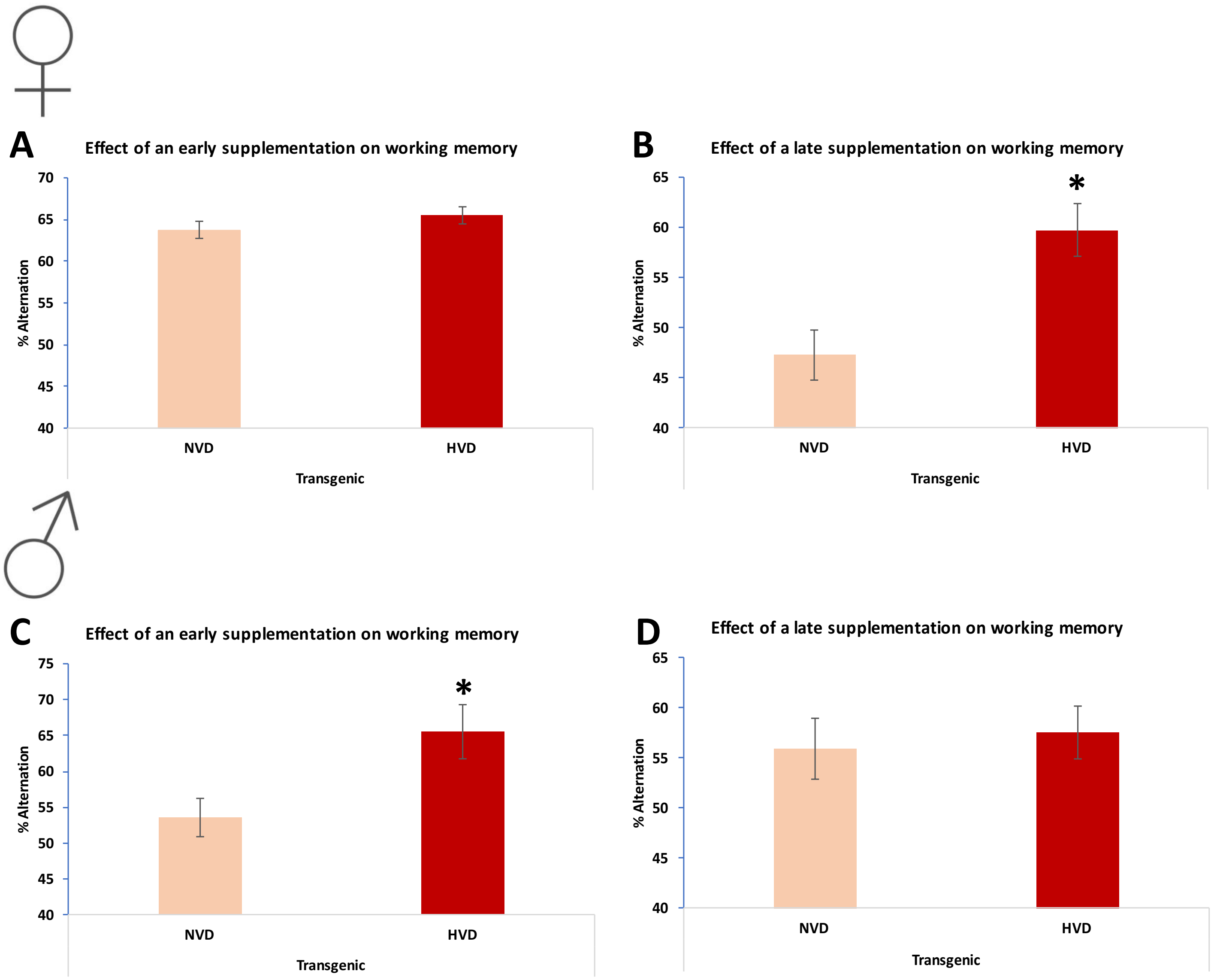
A
Effect of an early supplementation on working memory
% Alternation
70
65
60
55
50
45
40
NVD
HVD
Transgenic
B
Effect of a late supplementation on working memory
*
% Alternation
65
60
55
50
45
40
NVD
HVD
Transgenic
C
Effect of an early supplementation on working memory
*
% Alternation
75
70
65
60
55
50
45
40
NVD
HVD
Transgenic
D
Effect of a late supplementation on working memory
% Alternation
65
60
55
50
45
40
NVD
HVD
Transgenic
