# Supplementary material for: Vitamin D Improves Neurogenesis and Cognition in a Mouse Model of Alzheimer’s Disease
Source: Mol Neurobiol. 2018 Jan 9;55(8):6463–79. doi: 10.1007/s12035-017-0839-1 (PMC6061182; doi:10.1007/s12035-017-0839-1)
Supplement: Supplementary file 2 — Absence of in vitro effect of calcitriol on the differentiation of neuroblasts (neurospheres). A-B) E14,5 progenitor cells were cultivated in neurospheres, with or without calcitriol (n = 4) during 3 days. Cell differentiation was then evaluated by (A) shape modification and neurosphere adhesion to the well and (B) G1 cell cycle arrest during the treatment. C) VDR transcript expression was assessed using quantitative PCR in 3 day-old primary cultures of murine E14,5 progenitor cells and in 10 day-old primary cultures of murine neurons. The VDR is not transcribed neither in neuroblasts nor in neurons. (B, C). Cut off line corresponds to a theoretical DCT for a cDNA amplification at 30 cycles of PCR. (PDF 1436 kb) [file 12035_2017_839_MOESM2_ESM.pdf]

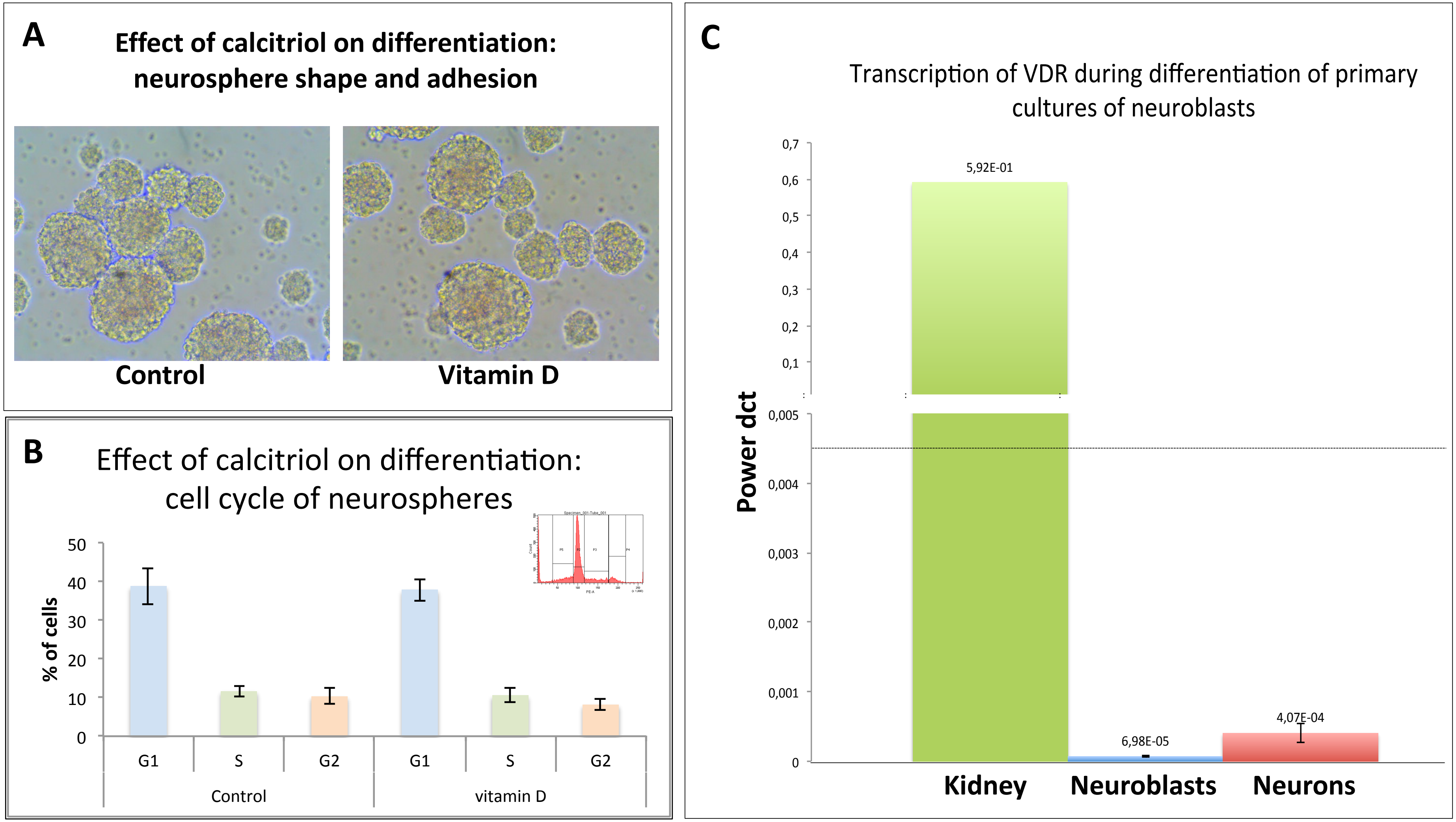
A
Effect of calcitriol on differentiation:
neurosphere shape and adhesion
Control
Vitamin D
B
Effect of calcitriol on differentiation:
cell cycle of neurospheres
% of cells
50
40
30
20
10
0
G1
S
G2
G1
S
G2
Control
vitamin D
C
Transcription of VDR during differentiation of primary
cultures of neuroblasts
Power dct
0,7
0,6
0,5
0,4
0,3
0,2
0,1
0,005
0,004
0,003
0,002
0,001
0
5,92E-01
6,98E-05
4,07E-04
Kidney
Neuroblasts
Neurons
