# Supplementary figures and images for: Vitamin D Improves Neurogenesis and Cognition in a Mouse Model of Alzheimer’s Disease
Source: Mol Neurobiol. 2018 Jan 9;55(8):6463–79. doi: 10.1007/s12035-017-0839-1 (PMC6061182; doi:10.1007/s12035-017-0839-1)

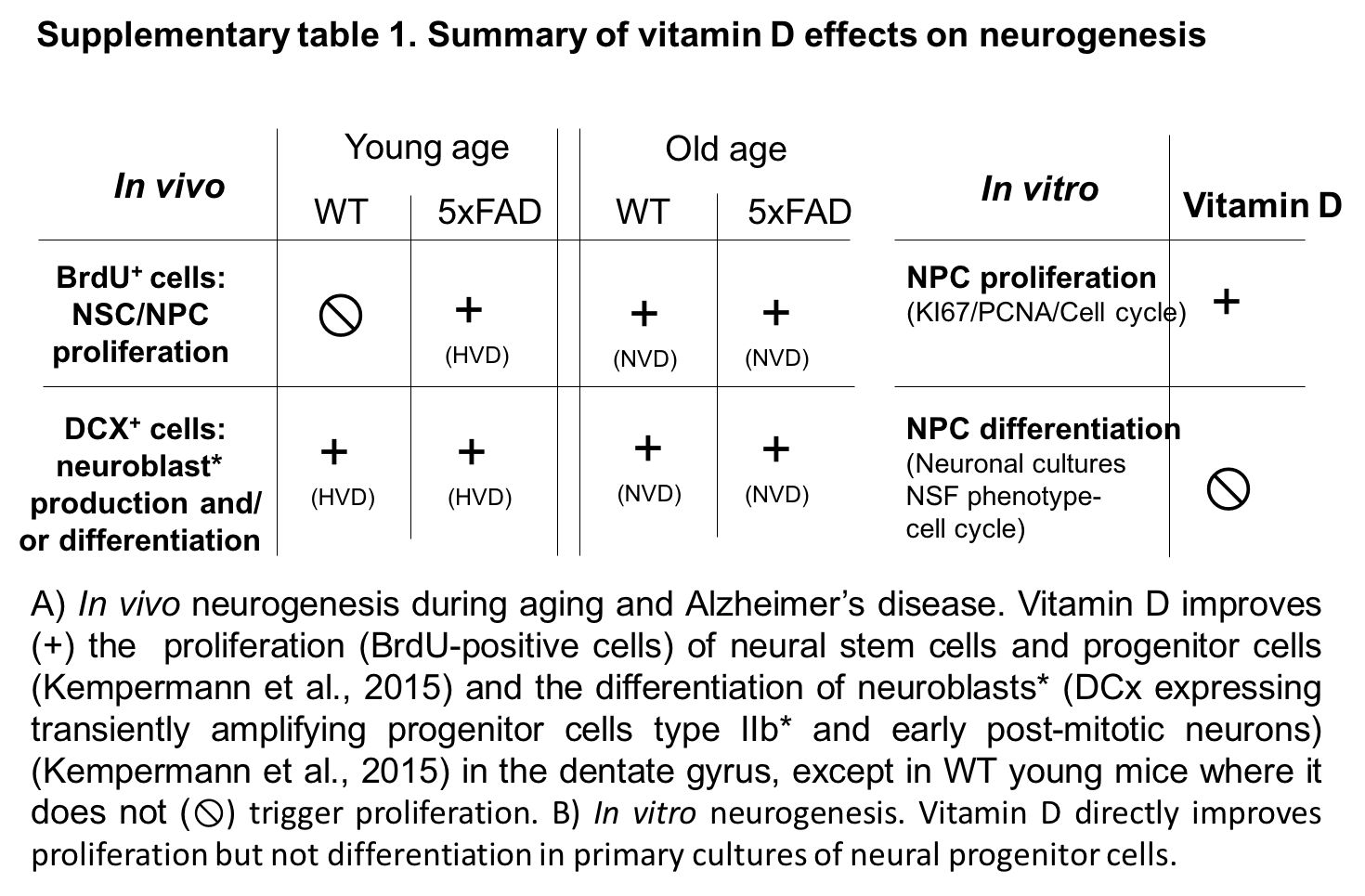

Supplement: Supplementary file 3 — (DOCX 207 kb) [file 12035_2017_839_MOESM3_ESM.docx]
